# LncRNA GACAT1 induces tongue squamous cell carcinoma migration and proliferation via miR‐149

**DOI:** 10.1111/jcmm.16690

**Published:** 2021-08-10

**Authors:** Xueling Wang, Zuode Gong, Long Ma, Qibao Wang

**Affiliations:** ^1^ Department of Stomatology Aerospace Center Hospital Beijing China; ^2^ Department of Endodontics Jinan Stomatological Hospital Jinan China

**Keywords:** GACAT1, miR‐149, tongue squamous cell carcinoma

## Abstract

Recent studies have observed that lncRNAs (long non‐coding RNAs) are involved in the progression of various tumours including tongue squamous cell carcinoma (TSCC). Recently, a new lnRNA, GACAT1, has been firstly identified in gastric cancer. However, its potential role in TSCC remains unknown. In this reference, we observed that GACAT1 was overexpressed in TSCC samples and cell lines. Of 25 TSCC specimens, GACAT1 expression was overexpressed in 18 patients (18/25, 72%) compared to non‐tumour specimens. Ectopic expression of GACAT1 induced cell growth and migration and promoted epithelial to mesenchymal transition in TSCC. In addition, ectopic expression of GACAT1 decreased miR‐149 expression in SCC1 cell. We observed that miR‐149 expression was down‐regulated in TSCC cell lines. Moreover, we observed that GACAT1 expression was negatively correlated with miR‐149 expression. GACAT1 overexpression induced TSCC cell growth and migration via regulating miR‐149 expression. These data provided that GACAT1 played an oncogenic role in the progression of TSCC partly through modulating miR‐149 expression.

## 1 INTRODUCTION

Tongue squamous cell carcinoma (TSCC) represents the most frequent type of oral squamous cell carcinoma (OSCC) and is famous for its high potential of metastasis and proliferation.^1^, ^2^, ^3^, ^4^, ^5^ TSCC usually leads to speech, deglutition and mastication malfunction.^6^, ^7^, ^8^ Despite the advancement in radiotherapy, chemotherapy and surgery, the 5‐year survival rate of this disease is still not satisfactory.^9^, ^10^, ^11^, ^12^ Survival rate improvement requires more understanding of initiation and progression of TSCC.^6^, ^13^, ^14^ Therefore, more works should be performed to explain the molecular mechanisms and process, which may identify new effective targets for TSCC treatment.

Long non‐coding RNAs (lncRNAs) are more than 200 nucleotides in length that can modulate gene expression in post‐transcriptional or transcriptional level.^15^, ^16^, ^17^, ^18^ Recent reports have showed that lncRNAs play essential roles in several cellular functions including cell development, growth, apoptosis, differentiation, invasion and migration.^19^, ^20^, ^21^, ^22^, ^23^ In addition, a number of studies suggested that lncRNAs were deregulated in various cancers such as colorectal cancer, breast cancer, osteosarcoma, hepatocellular carcinoma, bladder cancer and LSCC.^12^, ^24^, ^25^, ^26^, ^27^, ^28^ Recently, a new lnRNA, AC096655.1‐002 (GACAT1), was originally found to be correlated with progression of gastric cancer, and expression of GACAT1 was decreased in gastric cancer.^29^ Lower expression of CACAT1 was associated with TNM stages, lymph node metastasis, distant metastasis and differentiation. However, Shi et al^30^ reported that GACAT1 was overexpressed in gastric cancer samples and GACAT1 overexpression induced gastric cancer cell growth, migration and invasion. However, its role in TSCC development and the underlying molecular mechanisms has not been studied.

In the current study, our data observed that GACAT1 was overexpressed in TSCC samples and cell lines. Ectopic expression of GACAT1 induced cell growth and migration and promoted epithelial to mesenchymal transition (EMT) in TSCC.

## 2 MATERIAL AND METHODS

### 2.1 Clinical specimens

Samples of TSCC and their matched non‐tumour were collected from cases undergoing surgery in our department of Jinan Stomatological Hospital. These specimens were immediately frozen in the liquid nitrogen. Written consent of this study was collected from each patient, and this protocol was confirmed with Institutional Review Board of Jinan Stomatological Hospital (No. 20170203).

### 2.2 Cell cultured and transfection

Tca8113, SCC1, SCC‐4 and SCC‐15 TSCC cells were obtained from ATCC. These cells were kept in RPMI 1640 and penicillin, streptomycin and FBS. pcDNA3.1‐GACAT1 and control plasmid, miR‐149 mimic and scramble were collected from Shanghai GenePharma, and Lipofectamine 3000 (Invitrogen) was utilized for plasmid transfection.

### 2.3 Quantitative RT‐PCR

Total RNA of non‐tumour and TSCC samples, cells were purchased with TRIzol kit (Invitrogen) following to instructions. Quantitative RT‐PCR analysis was carried out to detect GACAT1, miR‐149 and ki‐67 expression using SYBR Green on the system of 7500 Real‐Time PCR (Applied Biosystems). U6 was utilized as control for miR‐149, and GAPDH was carried out as control for ki‐67 and GACAT1. The primers for GACAT1 were 5′‐ACCGGAGGAAAATCCCTAGC‐3′ (Forward) and 5′‐CCATAAAAGGGGCGGCTGT‐3′ (reverse); primers for miR‐149 were 5′‐CATCCTTTCTGGCTC CGTGT‐3′ (Forward) and 5′‐GCGTGATTCGTGCTCGTATATC‐3′ (reverse); primers for GAPDH were 5′‐TGTTCGTCATGGGTGTGAAC‐3′ (Forward) and 5′‐ATGGCATGGACTGTGGTCAT‐3′ (reverse). Ki‐67, 5′‐TCCTTTG GTGGGCACC TAAGACCTG‐3′ (Forward) and 5′‐TGATGGTT GAGGTCGT TCCTTGATG‐3′ (reverse). E‐cadherin, 5′‐CGAGAGCT ACACGTTCAC GG‐3′ (Forward) and 5′‐CGAGA GCTACACGTT CACGG‐3′ (reverse). Vimentin, 5′‐GACGCC ATCAACACC GAGTT‐3′ (Forward) and 5′‐CTTTGTCGTT GGTTAGCTGGT‐3′ (reverse).

### 2.4 Cell proliferation and migration assay

Cell growth rates were determined with MTT analysis (Sigma). Cell was kept in the 96‐well dish, and 10 μL was added to each well. After incubation for about 2 hours, the absorbance value at the 490 nm was read on the plate reader. For cell migration, wound scratch analysis was carried out. Pipette tip was utilized to create the cell wound. These treated cells were kept in the FBS‐free medium. Image of the wound was taken at the 0 and 48 hours.

### 2.5 Statistical analysis

Results were indicated as mean ± standard deviation. Student's *t* test was utilized to analysis the significance difference about these difference groups. The statistical analysis was carried out by SPSS *P* < .05 was defined as significance.

## 3 RESULTS

### 3.1 GACAT1 was overexpressed in TSCC samples and cell lines

**FIGURE 1:**
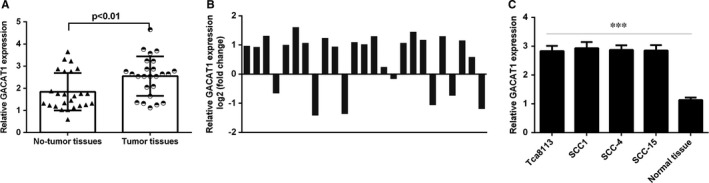
GACAT1 was overexpressed in TSCC samples and cell lines. A, The GACAT1 expression was detected by using qRT‐PCR analysis. B, Of 25 TSCC specimens, GACAT1 expression was overexpressed in 18 patients (18/25, 72%) compared to non‐tumour specimens. C, The GACAT1 expression in TSCC cell lines (Tca8113, SCC1, SCC‐4 and SCC‐15) and one non‐tumour specimen was detected by qRT‐PCR assay. ***P < .001

As indicated in Figure 1A, we proved that expression of GACAT1 was increased in TSCC specimens compared to non‐tumour specimens (Figure 1A). Of 25 TSCC specimens, GACAT1 expression was overexpressed in 18 patients (18/25, 72%) compared to non‐tumour specimens (Figure 1B). We observed that GACAT1 expression was overexpressed in TSCC cell lines (Tca8113, SCC1, SCC‐4 and SCC‐15) compared to non‐tumour specimen (Figure 1C).

### 3.2 MiR‐149 was down‐regulated in TSCC samples and cell lines

**FIGURE 2:**
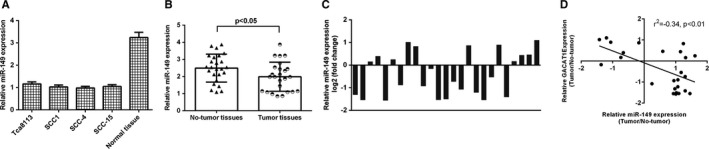
miR‐149 was down‐regulated in TSCC samples and cell lines. A, The miR‐149 expression in TSCC cell lines (Tca8113, SCC1, SCC‐4 and SCC‐15) and one non‐tumour specimen was detected by qRT‐PCR assay. B, The expression of miR‐149 was decreased in TSCC specimens compared to non‐tumour specimens. C, Of 25 TSCC specimens, miR‐149 expression was decreased in 14 patients (14/25, 56%) compared to non‐tumour specimens. D, GACAT1 expression was negative correlated with miR‐149 expression

We observed that miR‐149 expression was down‐regulated in TSCC cell lines (Tca8113, SCC1, SCC‐4 and SCC‐15) compared to non‐tumour specimen (Figure 2A). As shown in Figure 2B, we proved that expression of miR‐149 was decreased in TSCC specimens compared to non‐tumour specimens. Of 25 TSCC specimens, miR‐149 expression was decreased in 14 patients (14/25, 56%) compared to non‐tumour specimens (Figure 2C). Moreover, we observed that GACAT1 expression was negatively correlated with miR‐149 expression (Figure 2D).

### 3.3 GACAT1 induced TSCC cell growth and migration

**FIGURE 3:**
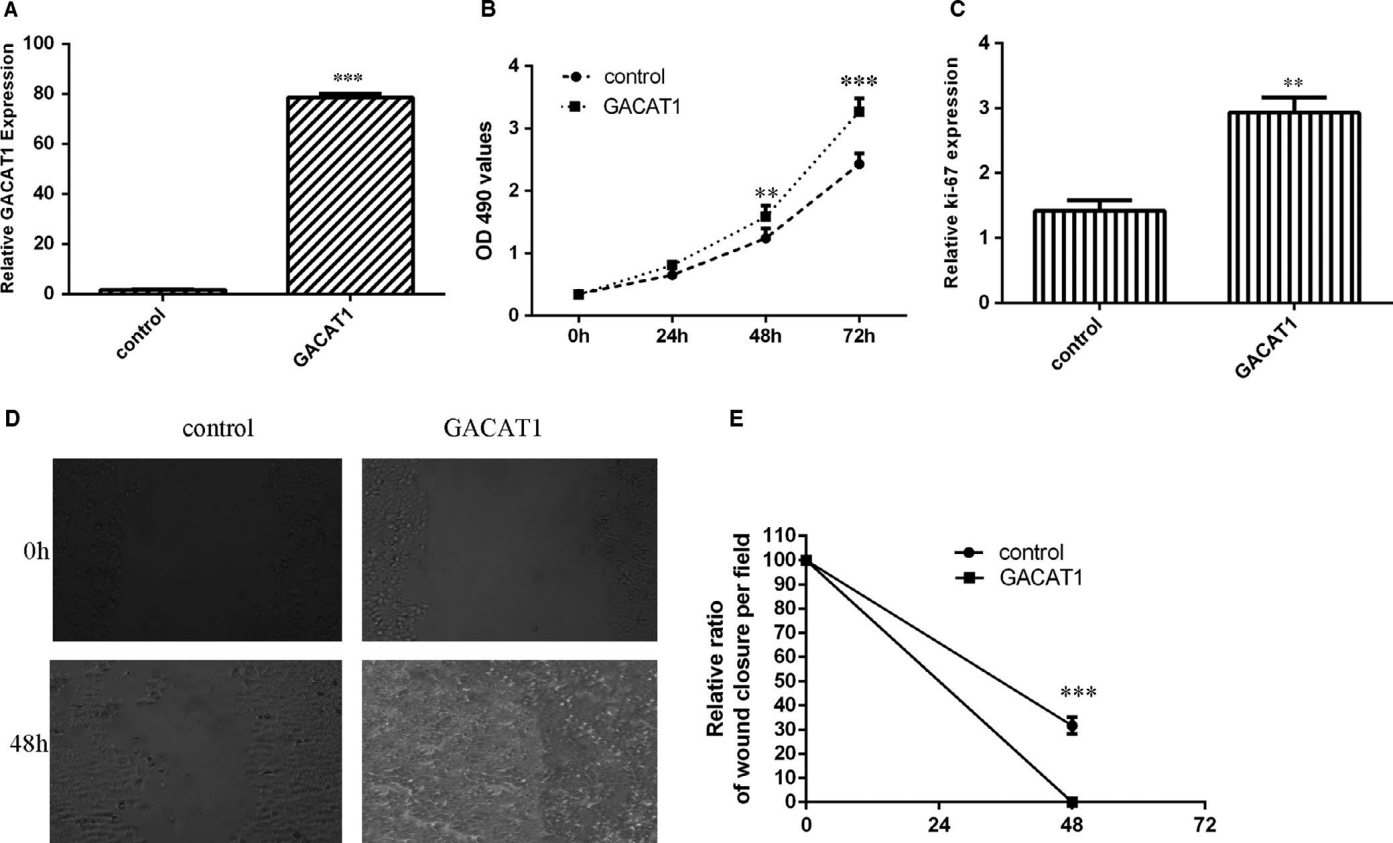
GACAT1 induced TSCC cell growth and migration. A, The expression of GACAT1 was determined by qRT‐PCR assay. B, Ectopic expression of GACAT1 enhanced SCC1 cell growth by using MTT assay. C, Overexpression of GACAT1 increased the ki‐67 expression in the SCC1 cell. D, GACAT1 overexpression promoted the SCC1 cell migration by using wound‐healing analysis. E, The relative wound closure was shown. **P < .01 and ***P < .001

To further investigate the function role of GACAT1 in TSCC development, we induced GACAT1 expression in SCC1 cell through transfection with pcDNA‐GACAT1 (Figure 3A). Ectopic expression of GACAT1 enhanced SCC1 cell growth by using MTT assay (Figure 3B). Overexpression of GACAT1 increased ki‐67 expression in SCC1 cell (Figure 3C). By using wound‐healing analysis, we observed that GACAT1 overexpression promoted SCC1 cell migration (Figure 3D,E).

### 3.4 GACAT1 promoted epithelial to mesenchymal transition in TSCC cell

**FIGURE 4:**
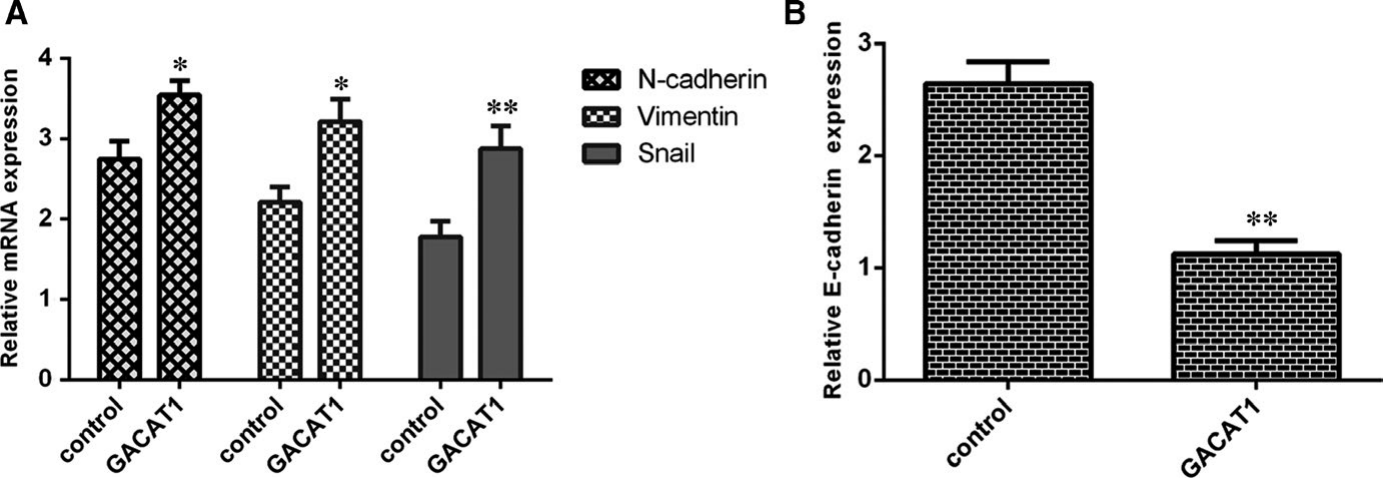
GACAT1 promoted epithelial to mesenchymal (EMT) transition in TSCC cell. A, Ectopic expression of GACAT1 induced the mesenchymal makers such as N‐cadherin, Vimentin and Snail expression in the SCC1 cell. B, Elevated expression of GACAT1 decreased epithelial maker E‐cadherin expression. *P < .05 and **P < .01

Next, we observed that ectopic expression of GACAT1 induced the mesenchymal makers such as N‐cadherin, Vimentin and Snail expression in the SCC1 cell (Figure 4A). Moreover, we indicated that elevated expression of GACAT1 decreased epithelial maker E‐cadherin expression (Figure 4B). These data suggested that GACAT1 overexpression induced EMT transition in TSCC.

### 3.5 GACAT1 suppressed miR‐149 expression in TSCC cell

**FIGURE 5:**
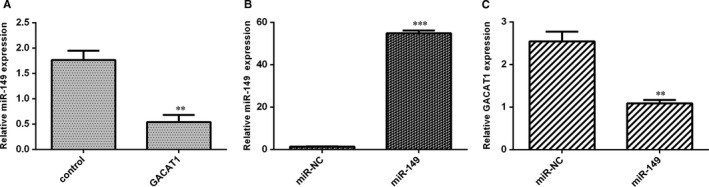
GACAT1 suppressed miR‐149 expression in TSCC cell. A, Ectopic expression of GACAT1 decreased miR‐149 expression in SCC1 cell. B, Expression of miR‐149 was significantly up‐regulated in the SCC1 cell via transfection with miR‐149 mimic. C, Elevated expression of miR‐149 suppressed the GACAT1 expression in SCC1 cell. **P < .01 and ***P < .001

Ectopic expression of GACAT1 decreased miR‐149 expression in SCC1 cell (Figure 5A). We observed that the expression of miR‐149 was significantly up‐regulated in the SCC1 cell via transfection with miR‐149 mimic (Figure 5B). Elevated expression of miR‐149 suppressed the GACAT1 expression in SCC1 cell (Figure 5C).

### 3.6 GACAT1 induced TSCC cell growth and migration via regulating miR‐149

**FIGURE 6:**
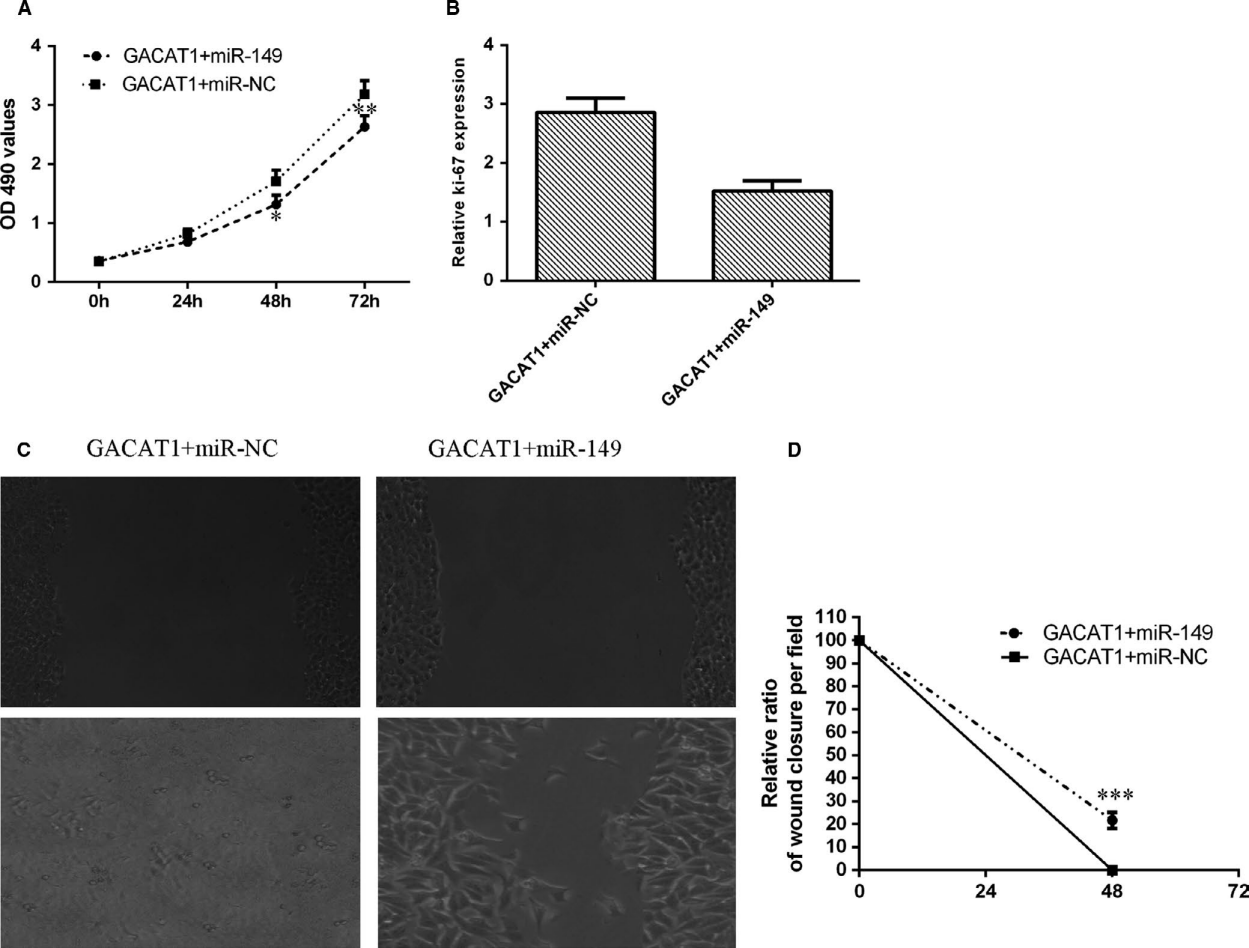
GACAT1 induced TSCC cell growth and migration via miR‐149. A, The cell proliferation was determined by using MTT assay. B, The mRNA expression of ki‐67 was measured by using qRT‐PCR analysis. C, The SCC1 cell migration in the different groups was measured by using wound‐healing analysis. D, The relative wound closure was shown. *P < .05; **P < .01 and ***P < .001

We next induced the expression of miR‐149 through transfection with miR‐149 mimic in the GACAT1‐overexpressing SCC1 cell. We observed that ectopic expression of miR‐149 decreased cell proliferation in the GACAT1‐overexpressing SCC1 cell (Figure 6A). We showed that elevated expression of miR‐149 suppressed ki‐67 expression in the GACAT1‐overexpressing SCC1 cell (Figure 6B). Moreover, we observed that miR‐149 overexpression decreased cell migration in GACAT1‐overexpressing SCC1 cell (Figure 6C,D).

## 4 DISCUSSION

In this study, we observed that GACAT1 was overexpressed in TSCC samples and cell lines. Of 25 TSCC specimens, GACAT1 was overexpressed in 18 patients (18/25, 72%) compared to non‐tumour specimens. Ectopic expression of GACAT1 induced TSCC cell growth and migration and promoted EMT in TSCC cell. In addition, ectopic expression of GACAT1 decreased miR‐149 expression in SCC1 cell. We observed that miR‐149 expression was down‐regulated in TSCC cell lines. Moreover, we observed that GACAT1 expression was negatively correlated with miR‐149 expression. GACAT1 overexpression induced TSCC cell growth and migration via regulating miR‐149 expression. These data provided that GACAT1 played an oncogenic role in the progression of TSCC partly through modulating miR‐149 expression.

Previous reports showed that lncRNAs acted critical roles in TSCC initiation, metastasis and growth. LncRNA H19 promoted TSCC invasion and migration through sponging miR‐let‐7.^31^ Knockdown of lncRNA TUC338 inhibited TSCC cell growth and increased cell apoptosis.^32^ LncRNA NKILA suppressed invasion and migration of TSCC cell through inhibiting epithelial‐mesenchymal transition.^33^ Recently, Zhong et al^34^ showed that GACAT1 was decreased in NSCLC tissues, and knockdown of GACAT1 inhibited cell growth and promoted apoptosis via suppressing miR‐422a. Wang et al^35^ indicated that GACAT1 induced breast tumour progression via sponging miR‐875‐3p. However, its role and mechanism in TSCC development have not been studied. We firstly studied the expression level of GACAT1 in TSCC samples and cell lines. We proved that expression of GACAT1 was increased in TSCC specimens and cells. Furthermore, we indicated that ectopic expression of GACAT1 induced TSCC cell growth and migration and promoted EMT in TSCC cell. However, more TSCC samples are needed in our further work. Thus, these data indicated that GACAT1 may act as one oncogene in tumorigenicity of TSCC cell and provided one potential therapeutic strategy for TSCC.

Increasing studies have suggested that lncRNAs function as ceRNA to modulate tumour progression.^36^, ^37^, ^38^ For instance, Ren and colleagues demonstrated that lncRNA CRNDE increased TSCC cell invasion and growth via down‐regulating miR‐384 expression.^39^ Zhang et al^40^ showed that KCNQ1OT1 modulated cisplatin resistance and proliferation through regulating miR‐211‐5p in tongue cancer. Ma et al^41^ reported that lncRNA GIHCG enhanced TSCC cell proliferation, cycle and migration via regulating miR‐429. Zuo and colleagues found that lncRNA CASC15 induced TSCC progression via modulating miR‐33a‐5p expression.^42^ In addition, previous report showed that GACAT1 overexpression induced gastric cancer cell proliferation, migration and invasion through modulating miR‐149 expression.^30^ In line with this, we found that ectopic expression of GACAT1 decreased miR‐149 expression in SCC1 cell. Moreover, GACAT1 overexpression induced TSCC cell growth and migration via regulating miR‐149 expression. Previous reports showed that miR‐149 played critical roles in tumour development. MiR‐149‐5p suppressed medullary thyroid carcinoma cell invasion and proliferation via targeting GIT1.^43^ Moreover, miR‐149 inhibited growth and induced sensitivity of ovarian tumour cell to cisplatin through targeting XIAP.^44^ MiR‐149 sensitizes oesophageal tumour cell to the cisplatin via regulating DNA polymerase β expression.^45^ Thus, our results suggested that GACAT1 promoted TSCC progression via modulating miR‐149 and its target genes.

Together, we described that GACAT1 was overexpressed in TSCC samples and cell lines. GACAT1 overexpression induced TSCC cell growth and migration via regulating miR‐149 expression. These data implied that GACAT1 played an oncogenic role in the progression of TSCC partly through modulating miR‐149 expression.

## CONFLICT OF INTEREST

There is no conflict of interest.

## AUTHOR CONTRIBUTIONS

**Xueling Wang:** Conceptualization (equal); Investigation (equal); Resources (equal); Software (equal); Writing‐original draft (equal); Writing‐review & editing (equal). **Zuode Gong:** Writing‐review & editing (equal). **Long Ma:** Software (equal); Writing‐review & editing (equal). **Qibao Wang:** Conceptualization (equal); Funding acquisition (equal); Writing‐review & editing (equal).

## Data Availability

Research data are not shared.

## References

[1] Wong TS, Liu XB, Wong BY, Ng RW, Yuen AP, Wei WI. Mature miR‐184 as potential oncogenic microRNA of squamous cell carcinoma of tongue. Clin Cancer Res. 2008;14:2588‐2592. 18451220 10.1158/1078-0432.CCR-07-0666

[2] Zheng D, Niu LX, Fei J, Li K, Tian JH. F‐18‐FDG PET/CT in diagnosis and staging of tongue squamous cell carcinoma. Int J Clin Exp Med. 2019;12:735‐743.

[3] Tam S, Amit M, Zafereo M, Bell D, Weber RS. Depth of invasion as a predictor of nodal disease and survival in patients with oral tongue squamous cell carcinoma. Head Neck. 2019;41:177‐184. 30537401 10.1002/hed.25506

[4] Guan C, Ouyang D, Qiao Y, et al. CA9 transcriptional expression determines prognosis and tumour grade in tongue squamous cell carcinoma patients. J Cell Mol Med. 2020;24(10):5832–5841. 32299152 10.1111/jcmm.15252 PMC7214172

[5] Haeggblom L, Nasman A, Ramqvist T, et al. TLR5 and TLR7 are differentially expressed in human papillomavirus‐positive and negative base of tongue squamous cell carcinoma, and TLR7 may have an independent prognostic influence. Acta Otolaryngol. 2019;139:206‐210. 30794027 10.1080/00016489.2018.1552014

[6] Jia B, Qiu XL, Chu HX, et al. Wnt7a predicts poor prognosis, and contributes to growth and metastasis in tongue squamous cell carcinoma. Oncol Rep. 2019;41:1749‐1758. 30747225 10.3892/or.2019.6974

[7] Suzuki S, Toyoma S, Tsuji T, Kawasaki Y, Yamada T. CD147 mediates transforming growth factor‐1‐induced epithelial‐mesenchymal transition and cell invasion in squamous cell carcinoma of the tongue. Exp Ther Med. 2019;17:2855‐2860. 30906472 10.3892/etm.2019.7230 PMC6425230

[8] De Paz D, Chang KP, Kao HK, et al. Clinical implications of tumor‐associated tissue eosinophilia in tongue squamous cell carcinoma. Laryngoscope. 2019;129:1123‐1129. 30098046 10.1002/lary.27413

[9] Kina S, Nakasone T, Kinjo T, Nimura F, Sunagawa N, Arasaki A. Outcomes after up‐front surgery and metronomic neoadjuvant chemotherapy with S‐1 or UFT for early tongue squamous cell carcinoma. Clin Oral Invest. 2019;23:2593‐2598. 10.1007/s00784-018-2689-2 30317400

[10] Shao BB, He LL. Hsa_circ_0001742 promotes tongue squamous cell carcinoma progression via modulating miR‐634 expression. Biochem Biophys Res Comm. 2019;513:135‐140. 30944081 10.1016/j.bbrc.2019.03.122

[11] Domingueti CB, Janini JBM, Paranaiba LMR, Lozano‐Burgos C, Olivero P, Gonzalez‐Arriagada WA. Prognostic value of immunoexpression of CCR4, CCR5, CCR7 and CXCR4 in squamous cell carcinoma of tongue and floor of the mouth. Med Oral Patol Oral. 2019;24:E354‐E363. 10.4317/medoral.22904 PMC6530956 31011147

[12] Li YR, Wan Q, Wang WW, et al. LncRNA ADAMTS9‐AS2 promotes tongue squamous cell carcinoma proliferation, migration and EMT via the miR‐600/EZH2 axis. Biomed Pharmacother. 2019;112:108719. 30970517 10.1016/j.biopha.2019.108719

[13] Yang X, Zhu JY, Dai YM, et al. Multi‐parametric effect in predicting tumor histological grade by using susceptibility weighted magnetic resonance imaging in tongue squamous cell carcinoma. BMC Med Imaging. 2019;19:24. 30866854 10.1186/s12880-019-0322-8 PMC6417004

[14] Zhang YL, Zhao FQ. MicroRNA‐758 inhibits tumorous behavior in tongue squamous cell carcinoma by directly targeting metadherin. Mol Med Rep. 2019;19:1883‐1890. 30628702 10.3892/mmr.2019.9805

[15] Zhao J, Gao Z, Zhang C, Wu H, Gu R, Jiang R. Long non‐coding RNA ASBEL promotes osteosarcoma cell proliferation, migration and invasion by regulating microRNA‐21. J Cell Biochem. 2018;119:6461‐6469. 29323740 10.1002/jcb.26671

[16] Zhao HX, Hou WG, Tao JG, et al. Upregulation of lncRNA HNF1A‐AS1 promotes cell proliferation and metastasis in osteosarcoma through activation of the Wnt/beta‐catenin signaling pathway. Am J Transl Res. 2016;8:3503‐3512. 27648140 PMC5009402

[17] Zhuang Y, Jiang HY, Li H, et al. Down‐regulation of long non‐coding RNA AFAP1‐AS1 inhibits tumor cell growth and invasion in lung adenocarcinoma. Am J Transl Res. 2017;9:2997‐3005. 28670387 PMC5489899

[18] Wei W, Liu Y, Lu YB, Yang B, Tang L. LncRNA XIST promotes pancreatic cancer proliferation through miR‐133a/EGFR. J Cell Biochem. 2017;118:3349‐3358. 28295543 10.1002/jcb.25988

[19] Wang OC, Yang F, Liu YH, et al. C‐MYC‐induced upregulation of lncRNA SNHG12 regulates cell proliferation, apoptosis and migration in triple‐negative breast cancer. Am J Transl Res. 2017;9:533‐545. 28337281 PMC5340688

[20] Wang G, Pan J, Zhang L, Wei Y, Wang C. Long non‐coding RNA CRNDE sponges miR‐384 to promote proliferation and metastasis of pancreatic cancer cells through upregulating IRS1. Cell Prolif. 2017;50:e12389. 10.1111/cpr.12389 PMC6529119 28940804

[21] Ma X, Qi S, Duan Z, et al. Long non‐coding RNA LOC554202 modulates chordoma cell proliferation and invasion by recruiting EZH2 and regulating miR‐31 expression. Cell Prolif. 2017;50:e12388. 10.1111/cpr.12388 PMC6529120 28963737

[22] Ma X, Li ZH, Li T, Zhu LWS, Li ZSN, Tian N. Long non‐coding RNA HOTAIR enhances angiogenesis by induction of VEGFA expression in glioma cells and transmission to endothelial cells via glioma cell derived‐extracellular vesicles. Am J Transl Res. 2017;9:5012‐5021. 29218099 PMC5714785

[23] Liao YW, Shen LF, Zhao HT, et al. LncRNA CASC2 interacts with miR‐181a to modulate glioma growth and resistance to TMZ through PTEN pathway. J Cell Biochem. 2017;118:1889‐1899. 28121023 10.1002/jcb.25910

[24] Li J, Lian YF, Yan CS, et al. Long non‐coding RNA FOXP4‐AS1 is an unfavourable prognostic factor and regulates proliferation and apoptosis in colorectal cancer. Cell Prolif. 2017;50:e12312. 10.1111/cpr.12312 PMC6529074 27790757

[25] Huan JL, Xing L, Lin QH, Xui H, Qin XJ. Long noncoding RNA CRNDE activates Wnt/beta‐catenin signaling pathway through acting as a molecular sponge of microRNA‐136 in human breast cancer. Am J Transl Res. 2017;9:1977‐1989. 28469804 PMC5411947

[26] Ye KS, Wang SK, Zhang HH, Han H, Ma B, Nan W. Long noncoding RNA GAS5 suppresses cell growth and epithelial‐mesenchymal transition in osteosarcoma by regulating the miR‐221/ARHI pathway. J Cell Biochem. 2017;118:4772‐4781. 28519068 10.1002/jcb.26145

[27] Deng L, Yang SB, Xu FF, Zhang JH. Long noncoding RNA CCAT1 promotes hepatocellular carcinoma progression by functioning as let‐7 sponge. J Exp Clin Cancer Res. 2015;34:18. 25884472 10.1186/s13046-015-0136-7 PMC4339002

[28] He A, Liu Y, Chen Z, et al. Over‐expression of long noncoding RNA BANCR inhibits malignant phenotypes of human bladder cancer. J Exp Clin Cancer Res. 2016;35:125. 27514530 10.1186/s13046-016-0397-9 PMC4981950

[29] Xiao BX, Guo JM. Long noncoding RNA AC096655.1‐002 has been officially named as gastric cancer‐associated transcript 1, GACAT1. Tumor Biol. 2013;34:3271. 10.1007/s13277-013-0916-7 23754450

[30] Shi XQ, Wang XQ, Hua YM. LncRNA GACAT1 promotes gastric cancer cell growth, invasion and migration by regulating MiR‐149‐mediated of ZBTB2 and SP1. J Cancer. 2018;9:3715‐3722. 30405842 10.7150/jca.27546 PMC6216017

[31] Kou N, Liu S, Li XJ, et al. H19 facilitates tongue squamous cell carcinoma migration and invasion via sponging miR‐let‐7. Oncol Res. 2019;27:173‐182. 29523225 10.3727/096504018X15202945197589 PMC7848458

[32] Ouyang K‐X, Zou R, Liang J, Bai Z, Li Z, Zhao J‐J. TUC338 overexpression leads to enhanced proliferation and reduced apoptosis in tongue squamous cell carcinoma cells in vitro. J Oral Maxillofac Surg. 2017;75:423–428. 27637778 10.1016/j.joms.2016.08.009

[33] Huang W, Cui XY, Chen JN, et al. Long non‐coding RNA NKILA inhibits migration and invasion of tongue squamous cell carcinoma cells via suppressing epithelial‐mesenchymal transition. Oncotarget. 2016;7:62520‐62532. 27613832 10.18632/oncotarget.11528 PMC5308743

[34] Zhong Y, Lin H, Li Q, Liu C, Zhong L. Downregulation of long non‐coding RNA GACAT1 suppresses proliferation and induces apoptosis of NSCLC cells by sponging microRNA‐422a. Int J Mol Med. 2021;47:659‐667. 33416153 10.3892/ijmm.2020.4826 PMC7797425

[35] Wang Q, Xue J, Ren Q, Li X, Qiu X. Long‐chain non‐coding RNA GACAT1 promotes development and progression of breast cancer by targeting microRNA‐875‐3p. Oncol Lett. 2020;19:2547‐2553. 32194758 10.3892/ol.2020.11260 PMC7039160

[36] Peng W, Si S, Zhang Q, et al. Long non‐coding RNA MEG3 functions as a competing endogenous RNA to regulate gastric cancer progression. J Exp Clin Cancer Res. 2015;34:79. 26253106 10.1186/s13046-015-0197-7 PMC4529701

[37] Liu X, Hou L, Huang W, Gao Y, Lv X, Tang J. The mechanism of long non‐coding RNA MEG3 for neurons apoptosis caused by hypoxia: mediated by miR‐181b‐12/15‐LOX signaling pathway. Front Cell Neurosci. 2016;10:201. 27642276 10.3389/fncel.2016.00201 PMC5009716

[38] Zhang J, Yao T, Wang Y, Yu J, Liu Y, Lin Z. Long noncoding RNA MEG3 is downregulated in cervical cancer and affects cell proliferation and apoptosis by regulating miR‐21. Cancer Biol Ther. 2016;17:104‐113. 26574780 10.1080/15384047.2015.1108496 PMC4847830

[39] Ren YX, He WT, Chen W, et al. CRNDE promotes cell tongue squamous cell carcinoma cell growth and invasion through suppressing miR‐384. J Cell Biochem. 2019;120:155‐163. 30242873 10.1002/jcb.27206

[40] Zhang SY, Ma HY, Zhang DM, et al. LncRNA KCNQ1OT1 regulates proliferation and cisplatin resistance in tongue cancer via miR‐211‐5p mediated Ezrin/Fak/Src signaling. Cell Death Dis. 2018;9:742. 29970910 10.1038/s41419-018-0793-5 PMC6030066

[41] Ma L, Wang QB, Gong ZD, Xue LD, Zuo ZB. Long noncoding RNA GIHCG enhanced tongue squamous cell carcinoma progression through regulating miR‐429. J Cell Biochem. 2018;119:9064‐9071. 29953645 10.1002/jcb.27164

[42] Zuo ZB, Ma L, Gong ZD, Xue LD, Wang QB. Long non‐coding RNA CASC15 promotes tongue squamous carcinoma progression through targeting miR‐33a‐5p. Environ Sci Pollut Res. 2018;25:22205‐22212. 10.1007/s11356-018-2300-z 29804249

[43] Ye X, Chen X. miR‐149‐5p inhibits cell proliferation and invasion through targeting GIT1 in medullary thyroid carcinoma. Oncol Lett. 2019;17:372‐378. 30655777 10.3892/ol.2018.9628 PMC6313157

[44] Sun L, Zhai R, Zhang L, Zhao S. MicroRNA‐149 suppresses the proliferation and increases the sensitivity of ovarian cancer cells to cisplatin by targeting X‐linked inhibitor of apoptosis. Oncol Lett. 2018;15:7328‐7334. 29731888 10.3892/ol.2018.8240 PMC5920966

[45] Wang Y, Chen J, Zhang M, et al. MiR‐149 sensitizes esophageal cancer cell lines to cisplatin by targeting DNA polymerase β. J Cell Mol Med. 2018;22:3857‐3865. 10.1111/jcmm.13659 PMC6050494 29726631

